# Knowledge of COVID-19 and Awareness of Physiotherapy Rehabilitation among Clinical Physiotherapy Students: A Cross-Sectional Study

**DOI:** 10.21315/mjms2022.29.6.11

**Published:** 2022-12-22

**Authors:** Premala Krishnan, Kamala Krishnan, Wei Kei Chan

**Affiliations:** Department of Physiotherapy, Faculty of Medicine and Health Sciences, Universiti Tunku Abdul Rahman, Selangor, Malaysia

**Keywords:** COVID-19, knowledge, awareness, physical therapy, rehabilitation, students

## Abstract

**Background:**

Physiotherapy rehabilitation improves patients’ activity in daily life and helps them return to work. Physiotherapy rehabilitation for COVID-19 patients mainly focuses on respiratory and functional rehabilitation assessment. This research project aims to assess the knowledge of COVID-19 and awareness of physiotherapy rehabilitation for COVID-19 patients among clinical physiotherapy students and the relationship between these variables.

**Methods:**

A preliminary, cross-sectional study was conducted on 159 clinical physiotherapy students from various education backgrounds. A three-part questionnaire assessing socio-demographic variables, knowledge of COVID-19 and awareness of physiotherapy rehabilitation for COVID-19 patients was distributed among clinical students from major physiotherapy programmes at tertiary institutions. Descriptive statistics, Chi-square tests and Spearman correlation tests were used for data analysis.

**Results:**

Most of the respondents (95.6%) were categorised as having an above average knowledge on COVID-19. Eighty-seven respondents (54.7%) were categorised as having an above average awareness of physiotherapy rehabilitation for COVID-19 patients. The knowledge of COVID-19 was positively correlated with awareness of physiotherapy rehabilitation (*P* < 0.05).

**Conclusion:**

This research study showed that the knowledge of COVID-19 and awareness level of physiotherapy rehabilitation in COVID-19 patients was above average among clinical physiotherapy students. The association between knowledge of COVID-19 and awareness of physiotherapy rehabilitation among clinical physiotherapy students had a weak positive correlation.

## Introduction

The whole world is currently facing the aftermath of the first surges of acute COVID-19 cases. Recent efforts are transitioning from finding a cure for COVID-19 to rehabilitating COVID-19 patients experiencing short-term or long-term effects. A prospective cohort study in Wuhan China reported that 40.64% of the discharged COVID-19 patients experienced symptoms such as cough, fatigue, tightness in chest, dyspnea and more; with pre-discharge physiotherapy, the incidence reported decreased to 13.74% within 3 weeks–4 weeks of discharge (1). Physiotherapists play a crucial role in assisting the early transfer of patients from the ICU to general wards, reviving a country’s health care system, reducing burden on the health care system and reducing medical expenses. This can help improve the social economy of nation by decreasing ICU cases and increasing hospital discharges (2). The importance of physiotherapy is further highlighted by the WHO and Pan American Health Organization releasing a comprehensive list of roles and guidelines for physiotherapy rehabilitation of acute COVID-19 patients called the ‘*Rehabilitation considerations during the COVID-19 outbreak*’ (3).

Physiotherapy rehabilitation provides a holistic approach in improving the daily activity of post COVID-19 patients and eventually prepares them to return to work. The respiratory and functional conditioning are focused on the rehabilitation component. The respiratory rehabilitation programme mainly consists of diaphragmatic breathing, pursed-lip breathing, positive expiratory pressure, incentive spirometry and respiratory muscle training. A 6-week rehabilitation programme for COVID-19 patients has shown notable improvement in lung function, functional capacity and quality of life (4). Functional rehabilitation, on the other hand, trains patients to regain functional independence and return to normal life as soon as possible. It includes active limb exercises, progressive muscle strengthening, neuromuscular electrical stimulation to strengthen muscles and gradual increase of daily activity (5).

The awareness of physiotherapy rehabilitation among future health care providers (current physiotherapy students) is vital for minimising short- and long-term effects of COVID-19, supporting nation’s health care system, and even improving a nation’s socioeconomic status during and after the pandemic. Due to a lack of surveys and research on the subject, this study aimed to assess awareness of physiotherapy rehabilitation among clinical physiotherapy students and to increase their awareness level on the importance of physiotherapy rehabilitation for COVID-19 patients. Thus, clinical physiotherapy students can apply and follow correct practices during their future clinical postings or other endeavors. Knowledge of transmission, prevention and control is important for physiotherapy students for the screening of patients and self-protection of the students since they may be directly involved with patients in post-COVID rehabilitation. A study among physiotherapy students in India shows adequate level of knowledge on COVID-19. It suggested improvement in the students’ knowledge of COVID-19 diagnosis and treatment strategies by organising educational programmes, webinars and awareness campaigns (6). With this recommendation as a basis, more studies should be carried out to assess knowledge and awareness among physiotherapy students and create an effective education system to prepare students to face COVID-19 rehabilitation.

## Methods

A structured 28-item questionnaire was administered. The questionnaire comprises three sections: i) socio-demographic information; ii) knowledge level of COVID-19 [adapted from (7)] and iii) awareness of physiotherapy rehabilitation, which was adapted based on guidelines provided by World Confederation for Physical Therapy, authorities of Association of Physical Therapy, Australian Physiotherapy Association and London North West University Healthcare (8). The questionnaire was developed in English and distributed using Google forms. The content of the questionnaire was validated by eight field experts and the content validity ratio was recorded at 0.79.

### Study Design and Settings

This cross-sectional study was conducted among clinical physiotherapy students from universities and colleges located in Klang Valley that provide diploma, degree or postgraduate courses in physiotherapy. A simple random sampling method was adopted. To minimise selection bias, this study used a representative sampling approach. The Google form was distributed via emails and social media platforms to students from all major tertiary institutions offering physiotherapy programmes.

### Study Participants

A total of 241 students submitted the Google form and early screening identified 200 forms as complete. These 200 students were then tested for eligibility. Forty-one students did not fit the inclusion criteria and the remaining 159 students were recruited into the study. Physiotherapy students who had attended clinical posting and consented to participate were included. Non-clinical students were excluded. The sample size of 237 was calculated based on the targeted population size of 1,900 with confidence interval set at 10% and confidence level at 95%. The response rate was recorded at 84%.

### Data Analysis

The data collected were entered into Microsoft Excel and exported to SPSS version 20.0 software. Descriptive statistics were used to analyse the demographic information. The relationship among the demographic data, knowledge and awareness was tested using Chi-square tests, where appropriate. A Spearman correlation test was used to find the relationship between knowledge level and awareness. A *P*-value of less than 0.05 was considered statistically significant.

## Results

### Sociodemographic Information

Of 159 students recruited, 115 (72%) were female. The majority participants (40%, *n* = 64) were from Year 3 followed by those from Year 4 (37%), Year 2 (13%), Year 1 (8%) and other clinical semesters (2%). Most of the responses were from students pursuing bachelor’s degree programmes (82%) followed by those from the Diploma in Physiotherapy programmes (10%) and Master or Doctor of Philosophy in Physiotherapy programmes (8%) (Table 1).

### Knowledge Level of COVID-19 among Clinical Physiotherapy Students

Most of the respondents (*n* = 152, 95.6%) were categorised as having an above average knowledge of COVID-19. The remaining seven respondents (4%) were categorised as having average knowledge of COVID-19 (Table 2).

### Awareness Level of Physiotherapy Rehabilitation in COVID-19 Patients

Over half of the respondents (54.7%) demonstrated an above average awareness level of physiotherapy rehabilitation in COVID-19 patients. About 44% of respondents fell into the average category and the remaining 1.3% into the below average category (refer Table 3).

### Relationship between Demographic Data, Knowledge Level and Awareness Level of Physiotherapy Rehabilitation

Level of study was strongly correlated with level of COVID-19 knowledge (*P* = 0.05). The relationship between knowledge on COVID-19 and awareness on physiotherapy rehabilitation for COVID-19 patients was assessed by using Spearman’s correlation coefficient. There was a weak but significant positive correlation between knowledge of COVID-19 and awareness of physiotherapy rehabilitation for COVID-19 patients (*P* < 0.05) (Table 4). Hence, respondents with more knowledge on COVID-19 were more likely to have more awareness on physiotherapy rehabilitation in COVID-19 patients (refer Table 4).

## Discussion

### Sociodemographic Information

The only sociodemographic variable that correlated with the knowledge level is the year of study of the respondents. An above average knowledge level was found in 40.8% of Year 3 clinical physiotherapy students followed by 37.5% of Year 4 students. This result is slightly different from that of a previous study done on medical students in IIUM, where the Year 4 and Year 5 students had higher knowledge than Year 3 students (9). However, the difference between the knowledge levels can possibly be due to more participation from Year 3 clinical physiotherapy students in this study. There were no associations between gender, programme enrolled, and cardiopulmonary postings and the knowledge of COVID-19 among respondents. This could be due to the equal quality of education provided to all clinical physiotherapy students by the universities or institutions.

### Knowledge Level of COVID-19 among Clinical Physiotherapy Students

Our results suggest that there is a misconception about transmission of COVID-19 among clinical physiotherapy students. Only 94 out of 159 respondents (59.1%) acknowledged that the virus cannot be transmitted through eating or contacting wild animals. This result is in line with those from IIUM and a study in India on medical students, where 57.1% and 55.9% of respondents had the same misconception, respectively (9, 10). However, the result is contrary to that of a study conducted among physiotherapy students in India, where only 9% of respondents had this misconception (6). A study from IIUM suggested that the false information from different sources might be the reason for confusion and misunderstanding among clinical physiotherapy students (9).

The knowledge about prevention and control of COVID-19 among respondents is high in the current study. The respondents were well informed that avoiding crowds (98.1%), giving correct treatment for COVID-19 patients (98.1%) and isolating from close contact for 14 days (98.7%) are effective ways to prevent and control the spread of the virus. This result is in line with studies from United Arab Emirates, Saudi Arabia and India (10–12). This is probably due to clinical posting experiences, mass media updates, law enforcement and better understanding by the respondents themselves (13, 14).

### Awareness Level of Physiotherapy Rehabilitation in COVID-19 Patients

Most of the respondents were aware of the effects and benefits of physiotherapy rehabilitation for COVID-19 patients. Among the respondents, 96.2% were aware that physiotherapy rehabilitation helps patients clear sputum, 96.5% were aware that it helps patients regain functional independence and improve quality of life and 81.1% were aware that rehabilitation such as endurance training reduces mortality rate and duration of hospital stays in COVID-19 patients.

Over half of the respondents were not aware of the contraindications and indications to conduct physiotherapy rehabilitation for COVID-19 patients. One hundred and eight respondents were not aware that COVID-19 patients with a dry and non-productive cough, lower respiratory tract condition and no exudative consolidation are contraindicated for pulmonary rehabilitation. Eighty-one respondents were not aware that exercise rehabilitation is recommended for ICU-admitted and ill patients. The reason for this result can be lack of experience with cardiopulmonary postings since only 70 (44%) out of 159 respondents had done cardiopulmonary clinical postings before. Not all the clinical physiotherapy students have the chance to participate in the management of COVID-19 since most of the COVID-19 patients were isolated in specific settings. Therefore, universities and institutions should emphasize education about physiotherapy rehabilitation specific for COVID-19 patients among clinical physiotherapy students to ensure that students are well informed on the relevant contraindications and indications.

### Correlation between Knowledge of COVID-19 and Awareness of Physiotherapy Rehabilitation on COVID-19 Patients

There was a weak positive correlation between knowledge of COVID-19 and awareness of physiotherapy rehabilitation for COVID-19 patients among respondents. According to a study assessing the knowledge and attitude of caregivers regarding rehabilitation provided to patients, improvement in rehabilitation measures occurred after increasing knowledge and attitude through a one-to-one training sessions. The training courses covered four aspects of rehabilitation: musculoskeletal, respiratory, gastrointestinal rehabilitation and deep vein thrombosis prevention. The rehabilitation education was given by distributing brochures, videos and more to caregivers. After the training sessions, significant improvement was noticed among caregivers of COVID-19 patients on the importance of four aspects of rehabilitation (15). This indicates that knowledge of COVID-19 is correlated to awareness of physiotherapy rehabilitation for COVID-19 patients.

### Limitations and Recommendations

COVID-19 is a new disease that presents the medical world with new challenges. Its pathophysiology and management are not completely understood yet. This study is limited to knowledge of COVID-19 and awareness of the role of physiotherapy but did not discuss any specific ‘gold standard’ rehabilitation protocols for COVID-19 patients. The small sample size and self-administered questionnaire may have drawn biases as well. The online-based random questionnaire distribution led to a low response rate mainly due to incomplete submissions of the questionnaire. A larger, face-to-face, population-based study testing knowledge and awareness of different COVID-19 clinical phases will be useful in understanding the condition and raising the awareness of the importance of physiotherapy rehabilitation.

## Conclusion

This study aimed to assess the knowledge about COVID-19 and awareness of physiotherapy rehabilitation for COVID-19 patients among future physiotherapists who will face post-COVID-19 situations in the coming years. It provides a channel for universities or institutions to understand physiotherapy students’ knowledge and awareness of COVID-19 and the importance of physiotherapy rehabilitation for COVID-19 patients. This study concludes that the level of COVID-19 knowledge and the level of awareness about physiotherapy rehabilitation for COVID-19 patients among clinical physiotherapy students is above average and that they are weakly correlated.

## Figures and Tables

**Table 1:** Demographic information

Characteristics	Frequency (*n*)	%
Gender		
Female	115	72.3
Male	44	27.7
Year of study		
Year 1	13	8.2
Year 2	21	13.2
Year 3	64	40.3
Year 4	59	37.1
Others	2	1.3
Level of study		
Diploma in Physiotherapy	15	9.4
Degree in Physiotherapy	131	82.4
Master or Doctor Philosophy in Physiotherapy	13	8.2
Attended cardiorespiratory posting		
Yes	70	44.0
No	89	56.0

**Table 2:** Knowledge on COVID-19 among clinical physiotherapy students

Statement	Correct response *n* (%)	Incorrect response *n* (%)
The main clinical symptoms of COVID-19 are fever, fatigue, dry cough and myalgia	151 (95)	8 (5)
Unlike the common cold, stuffy nose, runny nose, and sneezing are less common in persons infected with the COVID-19 virus	100 (62.9)	59 (37.1)
There currently is no effective cure for COVID-19, but early symptomatic and supportive treatment can help most patients recover from the infection	145 (91.2)	14 (8.8)
Not all persons with COVID-19 will develop to severe cases. Only those who are elderly, have chronic illnesses, and are obese are more likely to be severe cases	125 (78.6)	34 (21.4)
Eating or contact with wild animals would result in the infection by the COVID-19 virus	94 (59.1)	65 (40.9)
A person with COVID-19 cannot transmit the virus to others when a fever is not present	145 (91.2)	14 (8.8)
The COVID-19 virus spreads via droplets	147 (92.5)	12 (7.5)
Low risk people can wear general medical masks to prevent the infection by the COVID-19 virus	127 (79.9)	32 (20.1)
It is not necessary for children and young adults to take measures to prevent the infection by the COVID-19 virus	148 (93.1)	11 (6.9)
To prevent the infection by COVID-19, individuals should avoid going to crowded places	156 (98.1)	3 (1.9)
Isolation and providing the right treatment for the people who are infected with the COVID-19 virus are some of the effective ways to reduce the spread of the virus	156 (98.1)	3 (1.9)
Close contact with COVID-19 patients must be immediately isolated in a proper place for 14 days	157 (98.7)	2 (1.3)

Notes: *n* = sample size; (%) = percentage

**Table 3:** Awareness on physiotherapy rehabilitation for COVID-19 patients among clinical physiotherapy students

Statement	Correct response *n* (%)	Incorrect response *n* (%)
Physiotherapist helps COVID-19 patients to clear airways (remove sputum)	153 (96.2)	6 (3.8)
Respiratory muscle training and aerobic exercises are contraindicated for COVID-19 patients	127 (79.9)	32 (20.1)
COVID-19 patients who have dry and non-productive cough, present with lower respiratory tract condition and do not have exudative consolidation are contraindicated for pulmonary rehabilitation	51 (32.1)	108 (67.9)
Exercise rehabilitation is not recommended for COVID-19 patients who experienced ICU-acquired weakness, fragility, multiple comorbidities and old age	78 (49.1)	81 (50.9)
Physiotherapy rehabilitation helps COVID-19 patients to regain functional independence and improve quality of life	155 (96.5)	4 (2.5)
Physiotherapy rehabilitation such as exercise endurance training does not contribute in reducing mortality rate and duration of hospital stays among COVID-19 patients	129 (81.1)	30 (18.9)
Physiotherapy rehabilitation includes Active Cycle of Breathing Techniques (ACBT) such as breathing control, deep breathing exercise and huffing for COVID-19 patient	152 (95.6)	7 (4.4)
Breathing training such as abdominal breathing, pursed-lip breathing and thoracic expansion exercise, do not help COVID-19 patients to improve respiratory function	128 (80.5)	31 (19.5)
Physiotherapy rehabilitation includes Activity of Daily Living (ADL) training such as guide transfer, bathing, toileting, daily hygiene maintenance, and for weak COVID-19 patients	152 (95.6)	7 (4.4)
Following aerobic exercise plan is not applicable for COVID-19 patients who discharged from hospital or home isolated: start with low intensity exercise for minimum 6 weeks for five times per week, 30 min/day–60 min/day, increase intensity by 10% every week, to target 70% of the maximum heart rate is the limit of aerobic exercise	85 (53.5)	74 (46.5)

Notes: *n* = sample size; (%) = percentage

**Table 4:** Correlation between knowledge on COVID-19 and awareness on physiotherapy rehabilitation on COVID-19 patients (*n* = 159)

Variables	Knowledge	Awareness	*P*-value
Knowledge	1.184^a^	0.190^b^*	0.016^c^
Awareness	0.190^b^*	1.546^a^	

Notes:

a SD;

b correlation coefficient (r);

c *P-*value;

* Correlation is significant at the 0.05 level (2-tailed)
